# The rate of change in clinical indicators can predict the progression of hepatitis B virus-related acute-on-chronic preliver failure

**DOI:** 10.1097/MD.0000000000040246

**Published:** 2024-10-25

**Authors:** Jun Lu, Zhihui Tu, Zhen Zhang, Shumei Wang, Zhuoqi Liu, Xiaohui Lu, Jun Zhang, Daya Luo

**Affiliations:** a Department of Intensive Care Unit, Infectious Disease Hospital Affiliated to Nanchang University, Nanchang, Jiangxi, China; b Department of Biochemistry and Molecular Biology, School of Basic Medical Sciences, Jiangxi Medical College, Nanchang University, Nanchang, Jiangxi, China.

**Keywords:** acute-on-chronic preliver failure, disease progression, hepatitis B virus, novel model, rate of change

## Abstract

The objective of this study was to investigate the predictors and predictive model construction of the progression of HBV-Pre.Acute-on-chronic liver failure (ACLF), a total of 133 patients with HBV-Pre.ACLF was divided into the progressive group (52 patients) and the recovery group (81 patients) according to whether they progressed to ACLF or not. The clinical parameters N%, L%, PLT, ALT, TBiL, ALB, Cre, Na, NH3, CRP, AFP, prothrombin time (PT), international normalized ratio (INR), FIB, and their rate of change at baseline were analyzed in the 2 groups. The independent risk factors for HBV-Pre.ACLF progression was found by univariate and multivariate analyses, and a predictive model was constructed. The clinical parameters ALB, FIB, Na, combined alprostadil treatment and MELD, and MELD-Na scores at baseline were significantly different between the 2 groups (*P* <.05), while ALT, TBiL, Cre, CHE, NH3, N%, L%, PLT, INR, and PT were not significantly different (*P* >.05). The change rates of Na, CHE, PT, FIB, CRP, Cre, PLT, and the ratio after to before of N% were significantly different between the 2 groups (*P* <.05), while the change rates of ALT, TBIL, NH3, AFP, L%, and the ratio after to before of INR were not significantly different between the 2 groups (*P* >.05). Univariate and multivariate analyses showed that baseline ALB, Na, FIB, combined alprostadil therapy and the rate of change of Na and PLT were protective factors for disease progression, and the rate of change of PT, CRP, and the ratio after to before of N% were independent risk factors for disease progression. The novel model was LogitP = −6.051 + 4.049×ΔPT + 0.626×ΔCRP + 4.527×the ratio after to before N% and its area under the curve was 0.944 (95% confidence interval: 0.900**–**0.988) predicting progression of HBV-Pre.ACLF, and the best cutoff value was −0.22. The patients with a higher logitP score (> −0.22) had an increased risk for progression to ACLF (*P* <.05). The novel model logitP shows good predictive value for the disease progression of HBV-Pre.ACLF.

## 1. Introduction

Acute-on-chronic liver failure (ACLF) refers to a complex group of clinical syndromes characterized by coagulopathy, jaundice, ascites, with or without hepatic encephalopathy and other major clinical manifestations, and it is based on chronic liver disease with impaired liver function resulting from multiple etiologies.^[1]^ At present, the international diagnostic criteria of liver failure mainly include the liver failure diagnosis and treatment guidelines of Chinese Medical Association, the consensus of Asia-Pacific Association for the Study of the Liver (APASL) and the European Association for the Study of the Liver-Chronic Liver Failure (EASL-CLIF) collaboration standard.^[2]^ACLF has numerous etiologies, with nonviral factors such as alcohol, adiposity and autoimmunity predominating in European and American countries, whereas chronic infection with hepatitis B virus (HBV) predominates in our country. Hepatitis B virus-associated ACLF (HBV-ACLF) is a common type of liver failure in China. HBV/HCV co-infection can cause more serious chronic liver diseases.^[3]^ HIV/HBV co-infection accelerates the progression of chronic HBV to end-stage liver disease compared to chronic HBV mono-infection. HBV/HIV co-infection alters the natural history of hepatitis B and renders the antiviral treatment more complex.^[4]^ We had excluded these cases in the study. The basic population that has ACLF is large, the condition is often critical, medical care is difficult, and the case fatality rate is high. Liver transplantation, as the only effective method, faces certain limitations in clinical application due to the shortage of liver resources and high costs.^[5]^

At present, the pathogenesis of HBV-ACLF has not been fully elucidated, but researchers acknowledge that there is a transitional “golden window period” from onset to progression to liver failure, which is the “acute-on-chronic preliver failure, pre. ACLF.”^[5]^ Although there are some disputes about the etiology and clinical characteristics of ACLF, it is believed that the short-term mortality of advanced patients is significantly higher than that of early and middle stage patients, which confirms the importance of early diagnosis and early treatment to improve the prognosis of patients.^[6]^ There are patients with pre. ACLF whose liver function has the potential to reverse, with aggressive and effective interventions resulting in greater prognostic improvement.^[7]^ Current clinical research focuses on the prediction of short- and long-term outcomes in ACLF, including the chronic liver failure sequential organ failure assessment model simplified score CLIF-C OFs, MELD, MELD-Na and scoring systems such as the Chinese study group hepatitis B-related ACLF prognostic scores (COSSH-ACLFs). These scoring systems have some limitations in clinical use,^[7]^ and their predictive value for early disease progression in ACLF needs further validation. In the clinic, it is of great clinical importance to explore specific markers that can be found to accurately predict the progression of ACLF in the early stages of ACLF and actively adopt effective interventions to inhibit progression. Therefore, we searched for independent risk factors and constructed a prediction model of disease progression by analyzing the baseline clinical indicators of HBV-pre. ACLF and the rate of change in each clinical indicator during the development of the disease to identify progression cases early. This will provide a theoretical basis for early aggressive and effective interventions for HBV-ACLF, with a view to repress disease progression through early intervention and thus improve prognosis, which is of some clinical significance.

Study of Zhang Qian had reported that the rate of change in clinical indicators has a certain predictive value for the progression of the disease.^[8]^ Through the study of the rate of change in clinical indicators, important clues for the progression of HBV-pre.ACLF are expected. Indicators with high sensitivity and specificity can help identify the disease progression, which will provide important guidance for the early adoption of effective interventions for HBV- pre. ACLF. In this study, we dynamically analyze the clinical indicators and the rate of change of each indicator in patients with HBV-pre. ACLF to explore the risk factors for disease progression and aimed to establish a prediction model for disease progression is reported below.

## 2. Patients and methods

### 2.1. Research object

**Figure 1.**
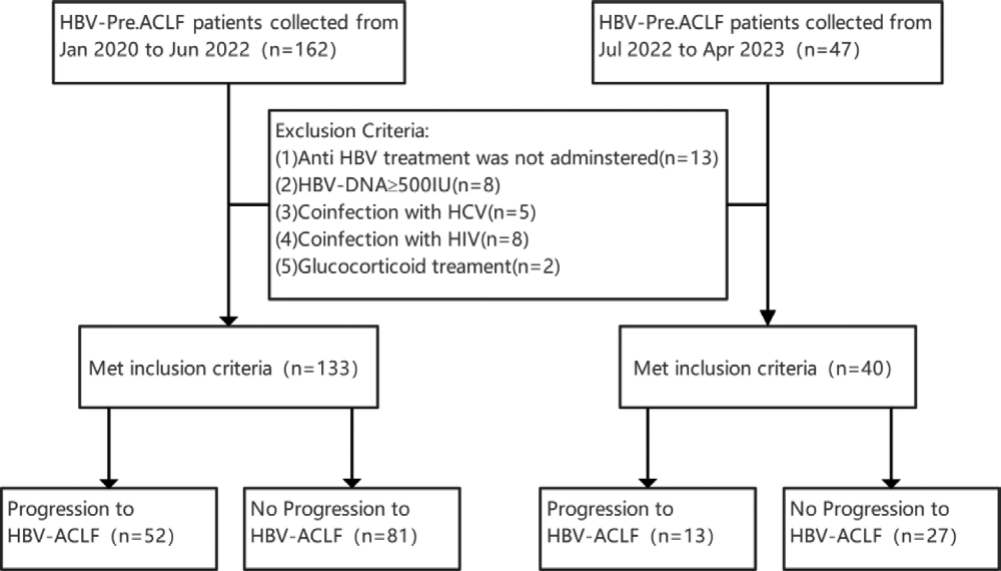
Outline of the screening and case enrollment protocol.

HBV-pre. ACLF were diagnosed as occurring on the basis of chronic non-cirrhotic liver disease according to our national guidelines “The Diagnosis and Treatment of Liver Failure (2018 Edition) .” The manifestations were as follows: ① extreme fatigue with severe gastrointestinal symptoms such as marked anorexia, vomiting and abdominal distension; ② substantial elevation of alanine transaminase (ALT) and/or aspartate transaminase (AST), progressive deepening of jaundice (85.5 ≤ TBiL < 171μmol/L) or daily rise ≥ 17.1 μmol/L; ③ bleeding tendency, 40%<PTA ≤ 50% (INR < 1.5).^[1]^All patients with HBV-related ACLF admitted to our hospital who were confirmed to be positive for HBsAg and HBV-DNA through hematology and immunology tests were selected. A total of 162 patients with a discharge diagnosis fulfilling the diagnostic criteria of HBV-related acute-on-chronic preliver failure (HBV-pre.ACLF) from January 2020 to June 2022 at Nanchang University Affiliated Infectious Disease Hospital were collected for this retrospective case–control study, and 29 patients were excluded for 1 or more of the following reasons: 7 had viral loads of more than 500 IU/mL at admission, and 11 did not choose anti-HBV treatment before admission, and 5 had co-infection with HCV, and 6 had co-infection with HIV. All patients were grouped according to disease development: 81 patients who did not progress to ACLF served as the recovery group, and 52 patients who progressed to ACLF served as the progression group. In addition, a total of 47 patients of HBV-pre.ACLF were collected from July 2022 to Apr 2023 as the test set at the same hospital to validate the model, and 7 patients did not meet the inclusion criteria (Fig. 1). In this study, HBV-pre.ACLF was diagnosed as the starting point of observation, and progressed to HBV-ACLF during hospitalization as the end point. The above information was obtained from the electronic medical record system of our hospital by recording the medical records, examination, and imaging results of the enrolled patients using case reports.

### 2.2. Inclusion and exclusion criteria

The inclusion criteria: HBV-pre.ACLF and HBV-ACLF were diagnosed as ACLF occurring on the basis of chronic non-cirrhotic liver disease according to our national guidelines “The Diagnosis and Treatment of Liver Failure (2018 Edition)”; antiviral use of nucleoside analogs and HBV-DNA negative status; and age range 18 to 65 years. The exclusion criteria were as follows:①Those not selected for anti-hepatitis B virus treatment; ②Human albumin, blood products and/or glucocorticoid treatment during observation period; ③Artificial liver support system treatment during hospitalization; ④Patients with concomitant viral hepatitis other than hepatitis B, alcoholic liver disease, autoimmune liver disease, drug-induced liver injury, Wilson disease, and comorbid tumors and other visceral serious diseases were excluded.

### 2.3. Research methods

The general conditions, clinical symptoms, examinations, imaging results, and treatment information of patients were obtained from the electronic case system and recorded in the experimental form. All the tests and imaging results were reviewed and confirmed by 2 experienced physicians. Clinical data collected on the enrolled patients included general basic information such as sex, age, white blood cell count (WBC), neutrophil count, percentage of neutrophils (N%),lymphocyte count, percentage of lymphocytes (L%), platelets (PLT), alanine aminotransferase (ALT), total bilirubin (TBiL), albumin (ALB), creatinine (Cre), serum sodium (Na), ammonia (NH_3_), C-reactive protein (CRP), and the clinical parameters of alpha-fetoprotein (AFP), prothrombin time (PT), normalized international ratio (INR), and fibrinogen (FIB), all of which were tested in the Department of Laboratory Medicine of our hospital, and these variables were selected for the study. Artificial liver and anti-hepatitis B virus therapy have significant effects on the progression of acute-on-chronic preliver failure.^[9]^ Therefore, information on combined alprostadil (PGE1), anti-hepatitis B virus therapy, and artificial liver therapy during the patients’ hospitalizations were also collected. Those cases whose hepatitis B virus loads exceeded 500IU/mL and did not receive anti-HBV treatment were excluded in the study. Clinical parameters and biochemical parameters at baseline were compared between the 2 groups. The rate of change in clinical indicators was calculated by reviewing the clinical indicator value minus the indicator value at baseline in addition to the number of days, using decimal places, and by taking a positive value when indicators increased and taking a negative value when decreased; the variations in some clinical indicators such as INR, N%, L% were evaluated by the ratio after to before. The ratio after to before is the ratio of the post value (after) to the pre value (before) of certain indicators. Statistical methods were used to analyze the differences in each indicator and its rate of change between the 2 groups to find the independent risk factors. Binary logistic regression analysis was used to construct a prediction model for HBV-pre.ACLF progression. The predictive abilities of the prediction models compared with each prognostic scoring system, including MELD, MELD-Na, CLIF-C OFs, and COSSH-ACLFs, on the progression of HBV-pre.ACLF were evaluated according to the area under the receiver operating characteristic curve (ROC). Each of the above scores was calculated according to the recording method in references.^[10–12]^

### 2.4. Instrument equipment

Liver function test reagents were purchased from Beijing Ninqiang Biotechnology Co., Ltd. and detected using a Hitachi 7600 automatic biochemistry instrument, and the detection method was velocity method. Coagulant function reagents were purchased from Hissen Mecon Biotech Co., Ltd. and detected using a Sysmexca-5100 automatic coagulometer, and the detection methods were optical coagulation and immunoturbidimetry method. Routine blood reagents were purchased from Shenzhen Mairui Bio Technology Co., Ltd., using a bc-6600plus hematology cell analyzer, and the detection methods were electrical impedance, flow cytometry, fluorescence staining and colorimetry method. All operations were performed according to the instrument and kit instructions.

### 2.5. Statistical analysis

Statistical analysis was performed using SPSS 26.0 statistical software (Chicago). The measurement data, first normal distribution test, and those that conformed to a normal distribution were expressed as the means ± (SD)using the t test from 2 independent samples. Nonnormal distributors are presented as median (quartiles) [M (P25, p75)] with the Mann–Whitney *U* test. Comparison of count data was performed using the *χ2* test; *P* < .05 was considered statistically significant. Variables with statistical differences were included in logistic binary regression analysis. Regression analysis using a binary logistic regression analysis was used to establish a predictive model; ROC curves were plotted to evaluate the predictive values of the prediction models. A comparison of the cumulative progression survival rates was conducted with the Kaplan–Meier method. Differences were considered statistically significant at *P* < .05.

### 2.6. Ethical statement

The study used medical records obtained from previous clinical consultations, conformed to medical ethical standards, met all of the following conditions and was exempted by the Ethics Committee of Nanchang Number 9 Hospital. The informed consents of all patients were obtained through the way of patient discharge follow-up. The ethics approval number was [2022] Lunjian Audition NO.(24).

1. The purpose of the study is clear.
2. The risk to the subjects of the study is not greater than the minimum risk;
3. The exemption of informed consent will not adversely affect the rights and health of the subjects;
4. The privacy and personally identifiable information of the subjects will be protected.

## 3. Results

### 3.1. General information of the patients in both groups

All patients with HBV-related ACLF admitted to our hospital who were confirmed to be positive for HBsAg and HBV-DNA through hematology and immunology tests were selected. A total of 162 HBV-pre.ACLF patients were enrolled from 2018.1 to 2022.6 Nanchang University Affiliated Infectious Disease Hospital, and 133 patients were finally screened out to meet the criteria according to the narrow criteria, and the study included 116 males and 17 females with a mean age of 44.34 ± 11.18 years. There were 81 patients in the recovery group, accounting for 60.9%, including 71 males and 10 females, with a mean age of 44.43 ± 11.45 years. There were 52 patients in the progression group, accounting for 39.1%, including 45 males and 7 females, with a mean age of 44.19 ± 10.87 years. There was no significant difference in general data between the 2 groups (*P* > .05). The clinical parameters ALT, TBiL, ALB, Cre, CHE, CRP, AFP, Na, NH_3_, INR, PT, FIB, N%, L%, PLT and MELD, and MELD-Na scores at baseline were compared between the 2 groups. The results showed no significant differences in ALT, TBiL, Cre, CHE, NH_3_, INR, PT, N%, L%, or PLT (*P* > .05). The ALB, FIB, Na, combined alprostadil treatment, and MELD, and MELD-Na scores were significantly different (*P* < .05) (Table 1).

**Table 1:** Comparison of general data and baseline levels between the 2 groups of HBV-Pre.ACLF patients.

Project	Recovery group (n = 81)	Progression group (n = 52)	Statistical value	*P* value
Gender (male/female)	71/10	45/7	0.00	>.05
Age (yr)	44.43 ± 11.45	44.19 ± 10.87	0.12	>.05
ALT (U/L)	597.8 (237.3, 1395.6)	547.90 (215.10, 1461.10)	0.24	>.05
TBil (μmol/L)	143.65 ± 28.77	149.02 ± 25.94	1.09	>.05
ALB (g/L)	36.00 ± 4.53	31.47 ± 3.83	5.96	<.05
CHE (U/L)	4860.23 ± 1588.39	4353.79 ± 1441.37	1.86	>.05
INR (IU)	1.55 ± 0.32	1.66 ± 0.32	1.83	>.05
PT (s)	18.37 ± 3.16	19.36 ± 2.17	1.96	>.05
FIB (g/L)	2.07 ± 0.75	1.79 ± 0.76	2.10	<.05
Cre (μmol/L)	72.58 ± 13.91	72.83 ± 20.29	0.08	>.05
Na (mmol/L)	138.47 ± 3.77	135.62 ± 3.67	4.31	<.05
NH_3_ (mmol/L)	33.00 (24.00, 43.00)	36.10 (25.30, 47.50)	0.69	>.05
N% (%)	66.19 ± 10.65	69.18 ± 10.60	1.58	>.05
L% (%)	22.58 ± 8.56	19.64 ± 8.35	1.95	>.05
PLT (10^9^/L)	111.50 (75.30, 147.80)	121.00 (90.00, 174.00)	1.34	>.05
CRP (mg/L)	8.90 (5.50, 14.00)	12.00 (7.90, 18.80)	0.24	>.05
AFP (U/L)	30.60 (8.50, 126.90)	24.50 (6.70, 72.00)	1.09	>.05
PGE1 therapy	57/24	20/32	13.23	<.05
MELD score	10.54 ± 3.83	12.65 ± 3.72	3.15	<.05
MELD-Na score	10.60 (7.80, 13.30)	12.90 (10.80, 16.90)	3.98	<.05

Abbreviations: AFP = alpha-fetoprotein, ALB = albumin, ALT = alanine aminotransferase, CHE = cholinesterase, Cre = creatine, CRP = C-reactive protein, FIB = fibrinogen, INR = international normalized ratio, L% = lymphocyte percentage, MELD = model for end-stage liver disease, MELD-Na = model for end-stage liver disease-sodium, N% = neutrophilic granulocyte percentage, Na = sodium, NH3 = ammonia, PGE1 therapy = alprostadil therapy, PLT = platelet, PT = prothrombin, TBil = total bilirubin.

### 3.2. Comparison of the change rate of clinical indicators between the 2 groups of HBV-pre. ACLF patients

Comparison of the change rates of the clinical parameters ALT, TBiL, ALB, CHE, Cre, CRP, AFP, Na, NH_3_, PT, FIB, PLT, and the ratio after to before of N%, L%, and INR at baseline between the 2 groups. The results showed no significant differences in the rates of change in ALT, TBiL, ALB, NH_3_, AFP, and the ratio after to before of L%, and INR (*P* > .05) and significant differences in the rates of change in CHE, Cre, CRP, Na, PT, FIB, PLT, and the ratio after to before of N% (*P* < .05) (Table 2).

**Table 2:** Comparison of the mean rate of change in each clinical indicator between the 2 groups of HBV-pre-AFCI patients.

Project	Recovery group (n = 81)	Progression group (n = 52)	Statistical value	*P* value
ΔALT	−31.63 (−70.69, −9.30)	−27.55 (−73.43, −12.20)	0.60	>.05
ΔTBil	2.40 (−0.85, 6.78)	4.20 (2.26, 6.03)	−1.88	>.05
ΔALB	0.22 (−0.38, 0.50)	−0.38 (−0.60, 0.49)	1.96	>.05
ΔCHE	85.40 (−116.50, 169.80)	−127.34 (−326.88, −71.65)	4.70	<.05
ΔPLT	3.40 (−2.04, 5.37)	−7.92 (−16.09, −4.36)	6.20	<.05
ΔPT	−0.33 (−0.47, 0.03)	0.62 (0.33, 0.92)	−7.73	<.05
ΔFIB	−0.01 (−0.09, 0.08)	−0.08 (−0.13, −0.05)	4.28	<.05
ΔCre	−1.60 (−3.52, 1.37)	1.47 (−1.65, 3.65)	−3.62	<.05
ΔCRP	−0.23 (−0.84, 0.45)	1.79 (0.98, 2.45)	−7.36	<.05
ΔAFP	−2.97 (−10.95, −0.27)	−1.53 (−9.70, 0.53)	−1.44	>.05
ΔNa	−0.40 (−0.63, 0.21)	−0.90 (−1.32, −0.43)	4.68	<.05
ΔNH3	1.00 (−3.10, 2.59)	2.10 (−0.83, 3.00)	−1.79	>.05
N% A/B ratio	1.02 ± 0.13	1.11 ± 0.16	3.33	<.05
L% A/B ratio	0.87 ± 0.17	0.81 ± 0.19	1.97	>.05
INR A/B ratio	0.99 ± 0.14	1.04 ± 0.17	1.66	>.05

Abbreviations: Δ = delta, A/B ratio = the ratio after to before, AFP = alpha-fetoprotein, ALB = albumin, ALT = alanine aminotransferase, CHE = cholinesterase, Cre = creatine, CRP = C- Reactive Protein, FIB = fibrinogen, INR = international normalized ratio, L% = lymphocyte percentage, N% = neutrophilic granulocyte percentage, Na = sodium, NH_3_ = ammonia, PLT = platelet, PT = prothrombin, TBil = total bilirubin.

### 3.3. Multivariate analyses of the progression of HBV-pre.ACLF

Binary logistic regression multivariate analyses was performed according to the selected parameters ALB, FIB, Na, and combined alprostadil treatment at baseline, and the rate of change of CHE, Na, Cre, PT, FIB, CRP, and the ratio after to before of N%. The rate of change of PT, CRP, Cre, and the ratio after to before of N% were independent risk factors for disease progression (Table 3).

**Table 3:** Logistic regression multivariate analysis of the progression of HBV-Pre.ACLF.

	*B*	Std. error	Vald	Sig	EXP(*B*)	95% CI for EXP(*B*)	
						Lower limit	Upper limit
ΔPT	4.154	0.980	17.948	0.000	63.677	9.320	435.081
ΔCre	0.112	0.086	1.672	0.196	1.118	0.944	1.324
ΔCRP	0.662	0.170	15.108	0.000	1.940	1.389	2.709
ΔNa	−0.236	0.534	0.196	0.658	0.789	0.277	2.249
N% A/B ratio	5.382	2.414	4.970	0.026	217.371	1.916	24654.679
PGE1 therapy	−0.763	0.721	1.122	0.290	2.145	0.522	8.807

Abbreviations: Δ = delta, ALB = albumin, CHE = cholinesterase, CI = credibility interval, Cre = creatine, CRP = C-reactive Protein, FIB = fibrinogen, N% = neutrophilic granulocyte percentage, N% A/B ratio = the ratio after to before of N%, Na = sodium, PGE1 therapy = alprostadil therapy, PLT = platelet, PT = prothrombin.

### 3.4. Predictive model construction

The results of binary logistic multivariate analysis showed that *P* > .05 of ΔCre, ΔNa, and alprostadil treatment, so they did not included in the model, and *P* < .05 of ΔPT, ΔCRP, and the ratio after to before of N% were included in the equation to construct a prediction model LogitP = −6.051 + 4.049*ΔPT + 0.626*ΔCRP + 4.527*the ratio after to before of N% for the progression of HBV-pre.ACLF (Table 4).

**Table 4:** Logistic regression equation.

	*B*	Std. error	Vald	Sig	EXP(*B*)	95% CI for EXP(*B*)	
						Lower limit	Upper limit
ΔPT	4.049	0.736	30.240	0.000	57.332	13.542	242.727
ΔCRP	0.626	0.158	15.624	0.000	1.870	1.371	2.551
N% A/B ratio	4.527	2.228	4.127	0.042	92.476	1.173	7292.238
Constant	−6.051	2.448	6.109	0.013	0.002		

Abbreviations: Δ = delta, CI = credibility interval, CRP = C-reactive protein, N% = neutrophilic granulocyte percentage, N% A/B ratio = the ratio after to before of N%, PT = prothrombin.

**Figure 2.**
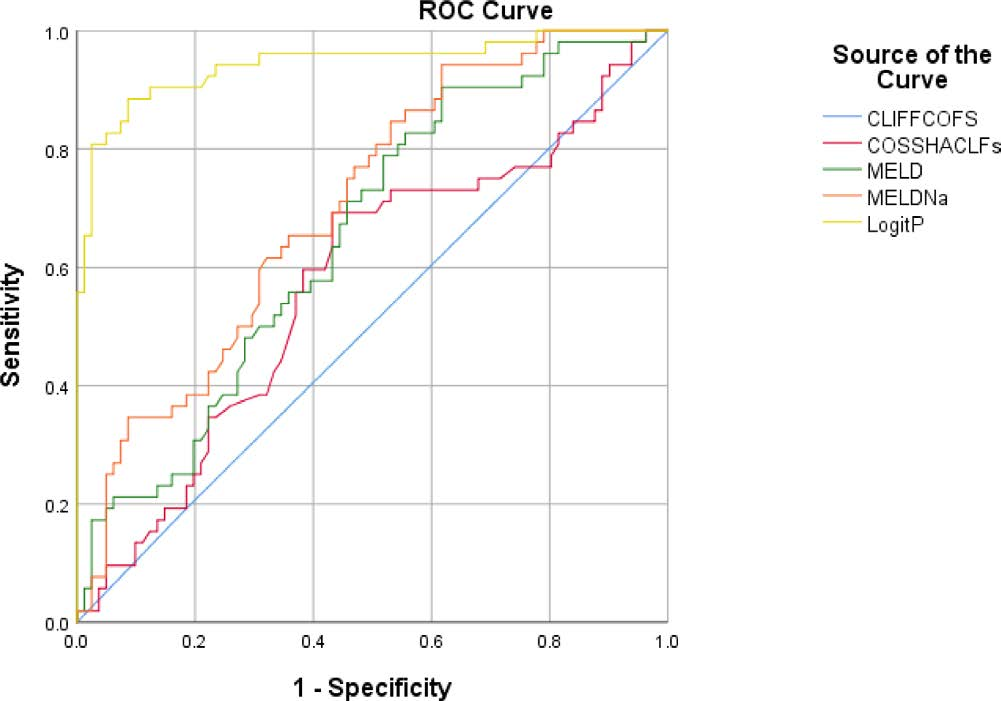
Comparison of the ROC curves of the various prognostic models with the novel model LogitP. The results showed that the new model LogitPhad the largest area under the curve. ROC = receiver operating characteristic.

Prediction model comparison of the area under the ROC curve between logitP and each prognostic scoring system for predicting HBV-pre.ACLF progression were conducted. The areas under the ROC curves of the scoring systems CLIF-C OFs, COSSH-ACLFs, MELD, and MELD-Na with the constructed predictive model logitP for predicting the progression of HBV-Pre.ACLF were 0.504, 0.576, 0.654, 0.705, and 0.944, respectively. The results illustrated that the LogitP score was superior to those models mentioned above (Fig. 2, Table 5).

**Table 5:** The area under the ROC for each prognostic scoring system versus model.

	AUROC	Std. error	Sig	EXP(B) 95% CI	
				Lower limit	Upper limit
CLIF-C OFs	0.504	0.052	0.945	0.403	0.605
COSSH-ACLFs	0.576	0.052	0.139	0.475	0.677
MELD scores	0.654	0.047	0.003	0.561	0.747
MELD-Na scores	0.705	0.045	0.000	0.617	0.792
LogitP	0.944	0.022	0.000	0.900	0.988

Abbreviations: AUROC = the area under the receiver operating characteristic, CI = credibility interval, CLIF-C Ofs = chronic liver failure consortium Organ Failure score, COSSH-ACLFs = Chines group on the study of severe hepatitis B-ACLFs, MELD = model of end-stage liver disease, MELD-Na = model of end-stage liver disease-sodium.

### 3.5. Validation of the new predictive model

**Figure 3.**
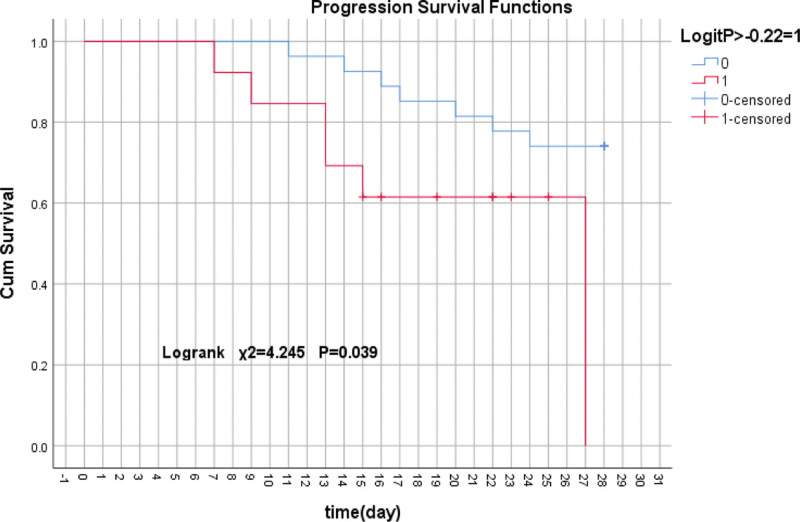
The patients with a higher LogitP score (> −0.22) had an increased risk for progression to acute-on-chronic liver failure (ACLF). The LogitP score demonstrated its applicability in predicting progression of HBV-pre.ACLF.

A cutoff point of the LogitP score −0.22 was suggested to indicate the progression of HBV-pre.ACLF, and it had an 88.5% sensitivity and 91.4% specificity. 40 patients of HBV-pre.ACLF in the test set were divided into 2 groups according to the best cutoff of LogitP score, 13 patients with a higher LogitP score (>−0.22) and 27 patients with lower LogitP score (≤−0.22). The progression survival rate at observation period were 46.2% (6/13) versus 25.9% (7/27) (*P* < .05) in groups of patients with LogitP score > −0.22 and ≤ −0.22, respectively. The patients with a higher LogitP score (>−0.22) had an increased risk for progression to ACLF (*P* < .05). Disease progression survival analysis was performed in both groups (Fig. 3).

## 4. Discussion

Acute-on-chronic liver failure is 1 of the leading causes of death in end-stage liver disease in China. There is a “gold window period” during the progression of the disease to ACLF, that is, acute-on-chronic preliver failure (Pre. ACLF), which is marked by a portion of patients with gradually improved recovery and a portion of patients with aggravation progressing to ACLF.^[5]^ Clinically, we cannot accurately determine whether a patient will progress to liver failure based on clinical features or laboratory and ancillary findings alone. In this study, we retrospectively analyzed the clinical data, laboratory clinical parameters and their dynamic changes in patients with HBV-Pre.ACLF to identify the independent risk factors and construct a prediction model for the progression of HBV-Pre.ACLF to liver failure.

Currently, several prognostic scoring systems are commonly used, such as the simplified score for the assessment of chronic liver failure sequential organ failure model (CLIF-C OFS), the model for end-stage liver disease (MELD), MELD-Na, and the Chinese study group on severe hepatitis B-related ACLF prognosis (COSSH-ACLFs) scoring system, which are mainly used to evaluate the short- and long-term outcomes of ACLF. Our results showed significant differences (*P* < .05) in the clinical and biochemical parameters ALB, FIB, Na, and MELD, and MELD-Na scores between the 2 groups when starting point baseline levels were observed, while ALT, TBiL, Cre, CHE, Na, N%, L%, PLT, INR, and PT were not significantly different between the 2 groups (*P* > .05), suggesting that some indicators such as ALB, FIB, and blood sodium at baseline may have some relationship with disease progression. When ACLF occurs, liver function is severely impaired, and the synthesis of body albumin and coagulation factors is reduced, resulting in lower ALB and coagulation dysfunction.^[13]^ Studies have confirmed that hyponatremia is 1 of the common complications of ACLF, which is closely related to the development and prognosis of the disease and can increase the incidence of complications such as hepatic encephalopathy and peritoneal effusion in patients with end-stage liver disease^.[14]^ The results of this study showed significant differences (*P* < .05) in the rate of change of clinical indicators CHE, PT, FIB, CRP, and PLT between the 2 groups, suggesting that the rate of change of some indicators such as CHE, PT, FIB, CRP, PLT, and the ratio after to before of N% may play some role in HBV-Pre.ACLF disease progression. CHE is mainly synthesized by the liver and is capable of reacting to hepatic synthetic function, and decreased levels of CHE in the blood suggest decreased hepatic synthetic function.^[15]^ The length of the PT depends on clotting factors, such as I, II, IV, V, VI, and VII, which are mainly synthesized by the liver, and clinically, the PT can respond to liver synthetic function. Patients with end-stage liver disease often present with coagulopathy and low platelet levels, and the severity of liver disease is closely related.^[16]^ Our results found a significant difference in the rate of change in CRP, and the ratio after to before of N% between the 2 groups (*P* < .05), indicating a possible association between CRP and N% with disease progression in HBV-Pre.ACLF. CRP, as an acute phase response protein, is able to respond to acute inflammatory activity, and its dynamic changes are of great value in the clinical diagnosis and prognosis judgment of inflammatory diseases.^[17]^ Many studies have confirmed that there are phases of systemic inflammation in ACLF in disease initiation and progression, and inflammatory responses play an important role in disease progression.^[18,19]^

In the present study, Logistic regression univariate and multivariate analyses of screened clinical indicators showed that baseline levels of ALB, Na, and FIB and whether or not they were combined with alprostadil treatment were protective factors for disease progression in HBV-Pre.ACLF, while the rate of change of PT, CRP, and the ratio after to before of N% were independent risk factors for its progression, which was compatible with the results of some current studies.^[20,21]^ We included the above independent risk factors in the binary logistic regression equation analysis and constructed a prediction model (logitP = −6.051 + 4.049*ΔPT + 0.626*ΔCRP + 4.527*the ratio after to before of N%) for HBV-Pre.ACLF progression. We applied the scoring system of CLIF-C OFs, COSSH-ACLFs, MELD, MELD-Na and the constructed prediction models logitP to predict the progression of HBV-pre.ACLF, and the areas under the ROC curves of them were 0.504, 0.576, 0.654, 0.705, and 0.944, respectively. The results showed that COSSH-ACLFs, MELD, and MELD-Na could predict the disease progression of HBV-Pre.ACLF, while CLIF-C OFs were less effective in predicting the progression of HBV-Pre.ACLF. Studies have reported that the COSSH-ACLFs scoring system has high predictive value for short- and long-term outcomes of HBV-ACLF, and our results showed its predictive efficacy for HBV-Pre.ACLF progression was not as good as its predictive efficacy for prognosis. The results showed that the area under the ROC curve of our new model logitP, which was constructed based on the rate of change of clinical indicators, was 0.944, showing high predictive efficacy for disease progression of HBV-Pre.ACLF.

It is believed that both the MELD, and MELD-Na scoring systems are better predictors of short-term outcomes in patients with ACLF, with similar predictive ability.^[22]^ The newly developed CHOSSH-ACLFs scoring system by Jun Li et al is more precise in the prognostic evaluation of HBV-ACLF patients than other scoring systems.^[23]^However, the predictive value of these prognostic scoring systems for disease progression in HBV-Pre.ACLF awaits further validation. This study applied multiple prognostic scoring systems to the prediction of HBV-Pre.ACLF progression, and although the areas under the curve predicted by these scoring systems all exceeded 0.5, none of them had overall predictive efficacy for the assessment of HBV-ACLF prognosis. This illustrates that in the clinic, there are certain limitations to more early prediction of HBV-ACLF disease development by prognostic scoring systems. This study focused on the early stage of HBV-ACLF onset, that is, HBV-Pre.ACLF by analyzing the clinical indicators of HBV-Pre.ACLF and their dynamic changes, in an effort to screen out HBV-ACLF patients earlier, timely and aggressive interventions should be taken to delay or even inhibit disease progression, thereby improving prognosis.

Moreover, the indicators included in these prognostic scoring systems were all direct detection values of indicators commonly used in the clinic, this study did not take into account situations such as the dynamic changes of indicators, and the dynamic changes of clinical indicators have more judgment value for the development of disease. Earlier studies reported that the rate of change in PT was not only able to respond to changes in liver function but also had some predictive value for HBV-ACLF prognosis.^[13]^ Our study found valuable predictors of disease progression by analyzing the relationship between the rate of change of each index and disease progression through dynamic monitoring of clinical indicators in HBV-Pre.ACLF patients. Our results showed that the rate of change in clinical indicators such as CHE, PT, FIB, CRP, PLT, and the ratio after to before of N% were closely related to the progression of the disease and were independent risk factors for the progression of HBV-Pre.ACLF. Some scholars have suggested that the inclusion of CRP in the MELD-Na scoring system could improve the prediction of mortality.^[24,25]^ Another study reported that early dynamic changes in CRP could serve as a predictive biomarker for the efficacy of the immune response.^[26]^ It also confirms that the dynamic changes in some clinical indicators are able to respond to the development process of the disease, which can prematurely predict the progression of the disease. In clinical practice, the parameter of PT is often used to evaluate the severity and prognosis of patients. This study found that the dynamic changes of PT, CRP and N% have a certain predictive value for the progression of the disease. Therefore, dynamic monitoring of the changes of these parameters is helpful to identify the progress of HBV-pre. ACLF. The parameters CRP and N% are closely related to infection, and they increase significantly when there is infection. In the clinic, we need to exclude the influence of factors such as infection, hemorrhagic disorders to improve the accuracy of the prediction, when we use this model.

In conclusion, HBV-ACLF has a rapid disease progression, making rescue and treatment difficult, with a high short-term case fatality rate, and early screening and intervention are essential. However, most studies on early warning of HBV-ACLF are prognostic evaluation systems, and few studies are early warning systems. These prognostic scoring systems at the early stage of HBV-ACLF disease occurrence did not have a high warning efficacy for the progression of the disease; therefore, developing an early warning system is clinically meaningful. Our prediction model logitP constructed based on the rate of change of clinical indicators PT, CRP, and the ratio after to before of N% showed better predictive value for HBV-Pre.ACLF disease progression.

The present study had the following deficiencies. First, the sample size included in this study was not large enough, and cases with incomplete and missing data encountered during the data collection process were filtered out, and more enrolled case data were needed for further analysis. Second, the present study was a single-center study case data, which remains to be validated by multi-center prospective cohort study data. In addition, the changing values of indicators PT, and FIB in this study were small, and the possibility of errors in the process of data analysis was large, which could easily cause some effects on the results.

## Author contributions

**Conceptualization:** Shumei Wang.

**Data curation:** Jun Lu, Zhihui Tu, Zhen Zhang, Xiaohui Lu.

**Formal analysis:** Jun Lu, Zhuoqi Liu.

**Funding acquisition:** Jun Lu, Zhen Zhang, Jun Zhang.

**Investigation:** Shumei Wang, Jun Zhang, Daya Luo.

**Methodology:** Jun Lu, Zhuoqi Liu, Daya Luo.

**Project administration:** Jun Zhang.

**Resources:** Daya Luo.

**Software:** Zhuoqi Liu.

**Supervision:** Zhen Zhang, Shumei Wang.

**Validation:** Zhen Zhang, Jun Zhang.

**Visualization:** Daya Luo.

**Writing – original draft:** Jun Lu, Zhihui Tu, Xiaohui Lu.

**Writing – review & editing:** Daya Luo.
